# Supplementary material for: Identification of 1600 replication origins in S. cerevisiae
Source: bioRxiv. 2023 Nov 25:2023.04.11.536402. Preprint. [Version 5] doi: 10.1101/2023.04.11.536402 (PMC10680564; doi:10.1101/2023.04.11.536402)
Supplement: 1 [file NIHPP2023.04.11.536402V5-supplement-1.pdf]

bioRxiv preprint doi: https://doi.org/10.1101/2023.04.11.536402; this version posted November 25, 2023. The copyright holder for this preprint (which was not certified by peer review) is the author/funder, who has granted bioRxiv a license to display the preprint in perpetuity. It is made available under aCC-BY 4.0 International license.

Figure S1. MCM-ChEC measurements are reproducible. 14 MCM-ChEC experiments show high reproducibility, with mean and median values of r^2 of 0.95 and 0.98, respectively. All plots show the same sample plotted on the x axes with each of the 13 remaining samples plotted on the y axes. 100 base pair windows centered on each of the most abundant 5,500 peaks of MCM-ChEC signal were quantified as the sum of per-base pair read depths, with all samples normalized to the same number of total counts for the entire genome. r^2 values are noted on each plot.

bioRxiv preprint doi: https://doi.org/10.1101/2023.04.11.536402; this version posted November 25, 2023. The copyright holder for this preprint (which was not certified by peer review) is the author/funder, who has granted bioRxiv a license to display the preprint in perpetuity. It is made available under aCC-BY 4.0 International license.

Figure S2. Replication activity and licensing at Mcm binding sites, assessed by ssDNA generation, BrdU incorporation, and qPCR.

(A) Average replication profiles, as determined by generation of ssDNA, for groups of 200 from the 1600 most prominent peaks of MCM-ChEC signal, centered on peak midpoints. WT (top row) and *rad53* (bottom row) cultures were arrested in G1 and released into medium containing HU. Samples were collected at 30 minutes, 1 hour, 2 hours and 3 hours and ssDNA was quantified, as described (Feng et al. 2006). (B) Average replication profiles, as determined by incorporation of BrdU, for groups of 200 from the 1600 most prominent peaks of MCM-ChEC signal, centered on peak midpoints. *rad53* cells were arrested in G1 and released into medium containing HU and BrdU. BrdU was quantified on Affymetrix chips. y axis shows average of log 2 of measurement/mean. (C) qPCR across origins using Mcm-ChEC-cut templates can be used to assess licensing, because increased cutting by Mcm-MNase is reflected in the requirement for increased qPCR cycles to generate a diagnostic product (Foss et al. 2021). Linear regression using qPCR at 7 origins allowed us to determine that the CMBS with rank 100 is licensed at 69% (green lines), and thereby to infer that the CMBSs in the 8th cohort, whose median CMBS abundance is 1.5% of that of the first cohort, is licensed at approximately 1% (69% x 0.015 = 1.0%). ARSs analyzed correspond to the following CMBS ranks, and were licensed at the indicated percentages: ARS111: rank 5, 76%; ARS1235: rank 75, 63%; ARS802: rank 183, 70%; ARS1427: rank 51, 76%; ARS1406: rank 42, 70%; ARS224: rank 9, 84%; ARS1103: rank 3, 90.0%.

bioRxiv preprint doi: https://doi.org/10.1101/2023.04.11.536402; this version posted November 25, 2023. The copyright holder for this preprint (which was not certified by peer review) is the author/funder, who has granted bioRxiv a license to display the preprint in perpetuity. It is made available under aCC-BY 4.0 International license.

Figure S3. Figure identical to Figure 3, except that nucleosomes were identified by MNase treatment of chromatin. Heat map showing nucleosomes flanking CMBSs is derived from library fragments in the 151-200 base pair range.

bioRxiv preprint doi: https://doi.org/10.1101/2023.04.11.536402; this version posted November 25, 2023. The copyright holder for this preprint (which was not certified by peer review) is the author/funder, who has granted bioRxiv a license to display the preprint in perpetuity. It is made available under aCC-BY 4.0 International license.

Figure S4. Comparison of pattern of GC skew when centered on ACSs versus CMBSs. (A) GC skew for 400 base pair sequences centered on 187 ACSs, oriented according to directionality specified in SGD. (B) GC skew for 400 base pair sequences centered on 5,500 most abundant CMBSs. GC skew is measured as in Figure 6.

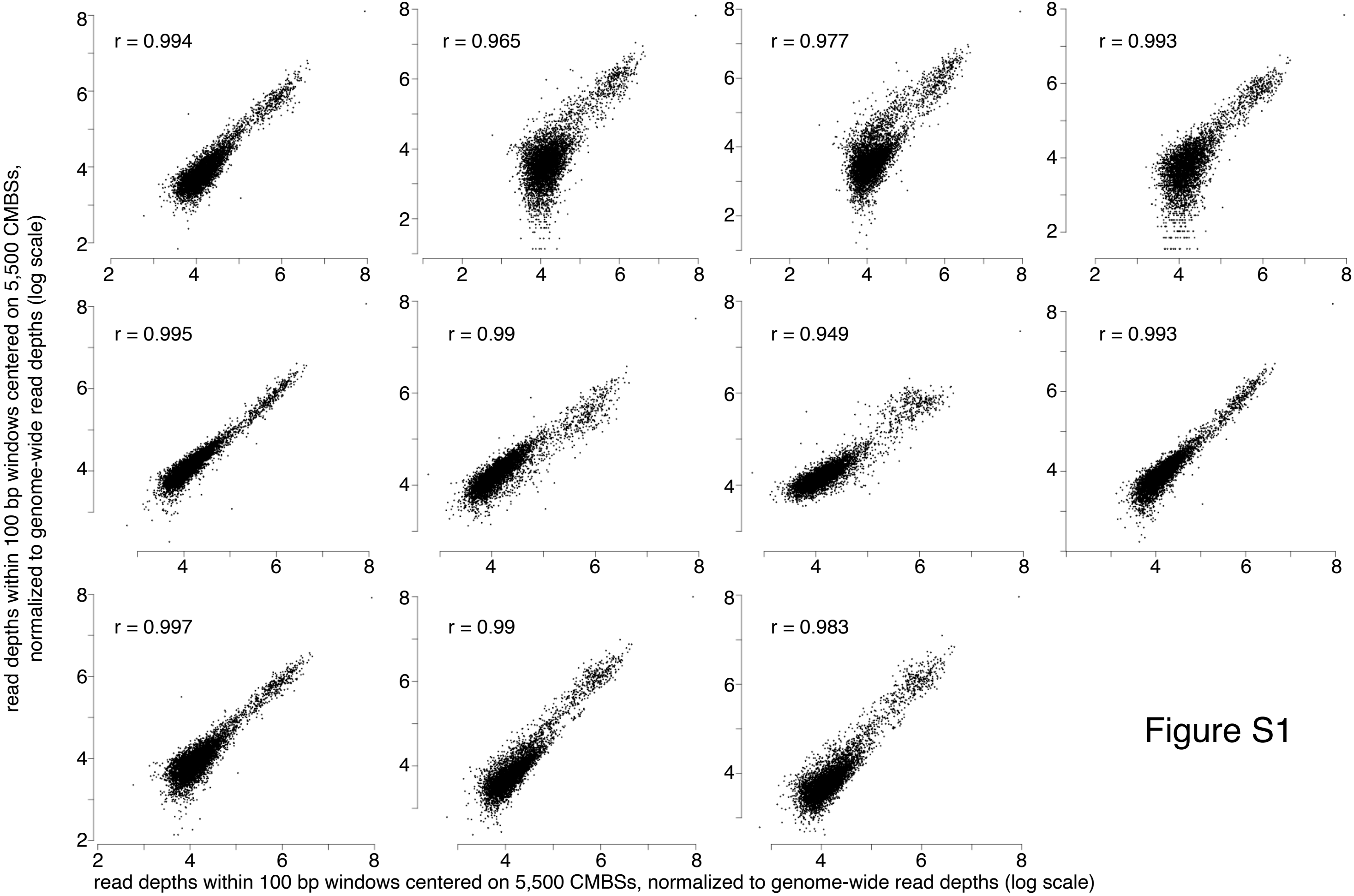
r = 0.994
r = 0.965
r = 0.977
r = 0.993
r = 0.995
r = 0.99
r = 0.949
r = 0.993
r = 0.997
r = 0.99
r = 0.983
read depths within 100 bp windows centered on 5,500 CMBSs, normalized to genome-wide read depths (log scale)
read depths within 100 bp windows centered on 5,500 CMBSs, normalized to genome-wide read depths (log scale)

Figure S1

Figure S2

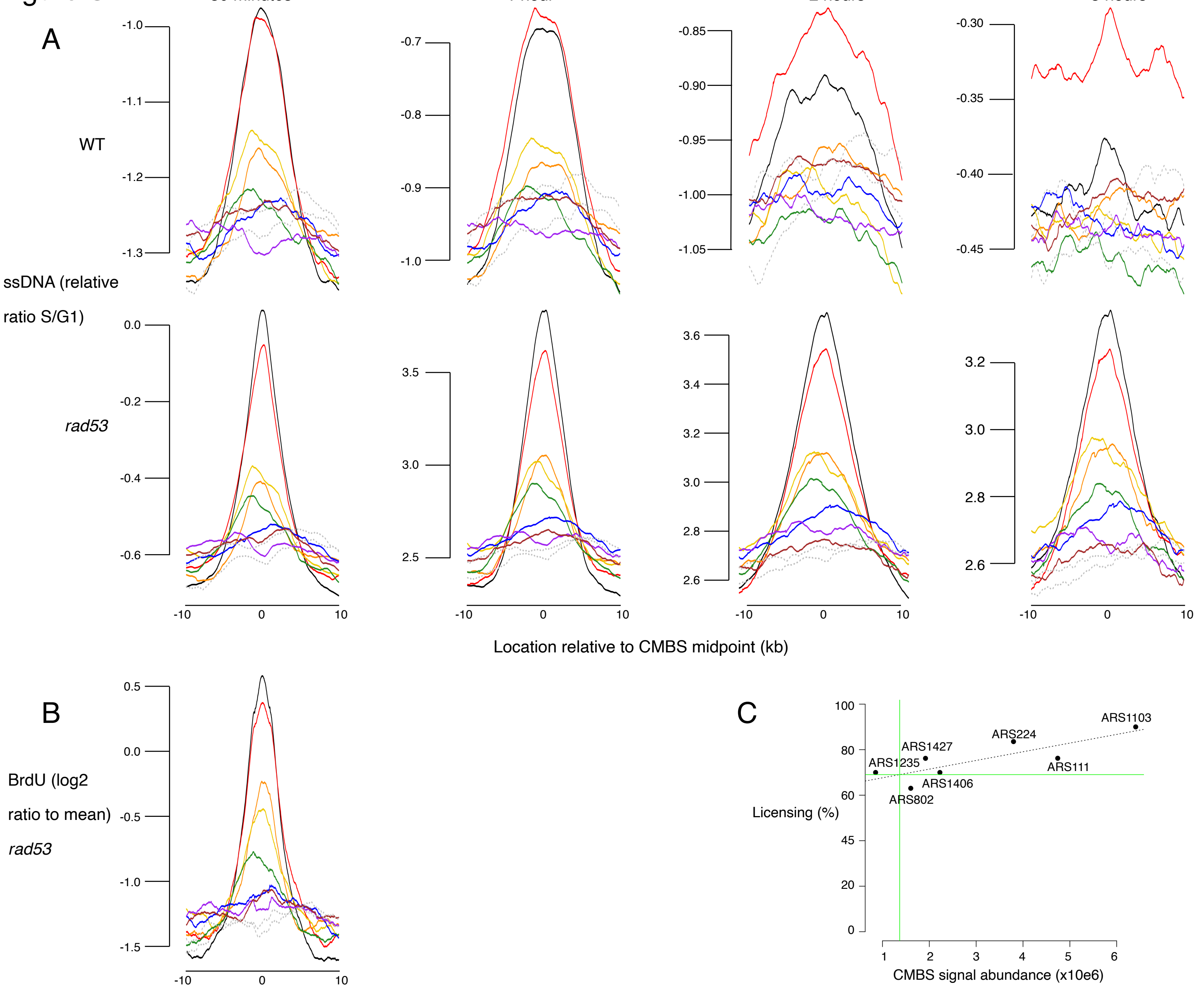

A
B
C
30 minutes
1 hour
2 hours
3 hours
WT
rad53
ssDNA (relative ratio S/G1)
BrdU (log2 ratio to mean)
Location relative to CMBS midpoint (kb)
Licensing (%)
CMBS signal abundance (x10e6)
ARS1103
ARS224
ARS1427
ARS1235
ARS111
ARS1406
ARS802

available under aCC-BY 4.0 International license.

Figure S3

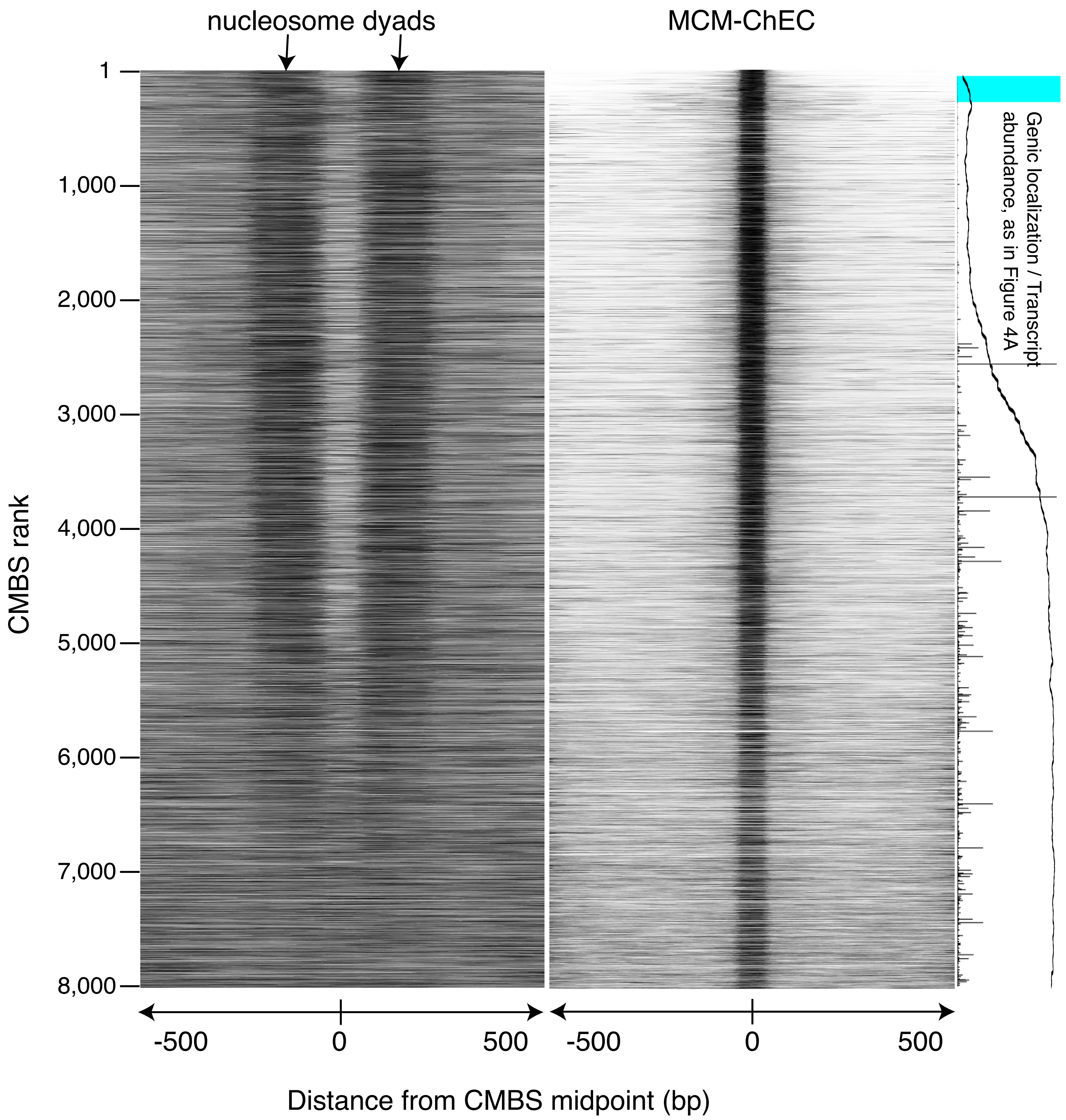

Figure S4

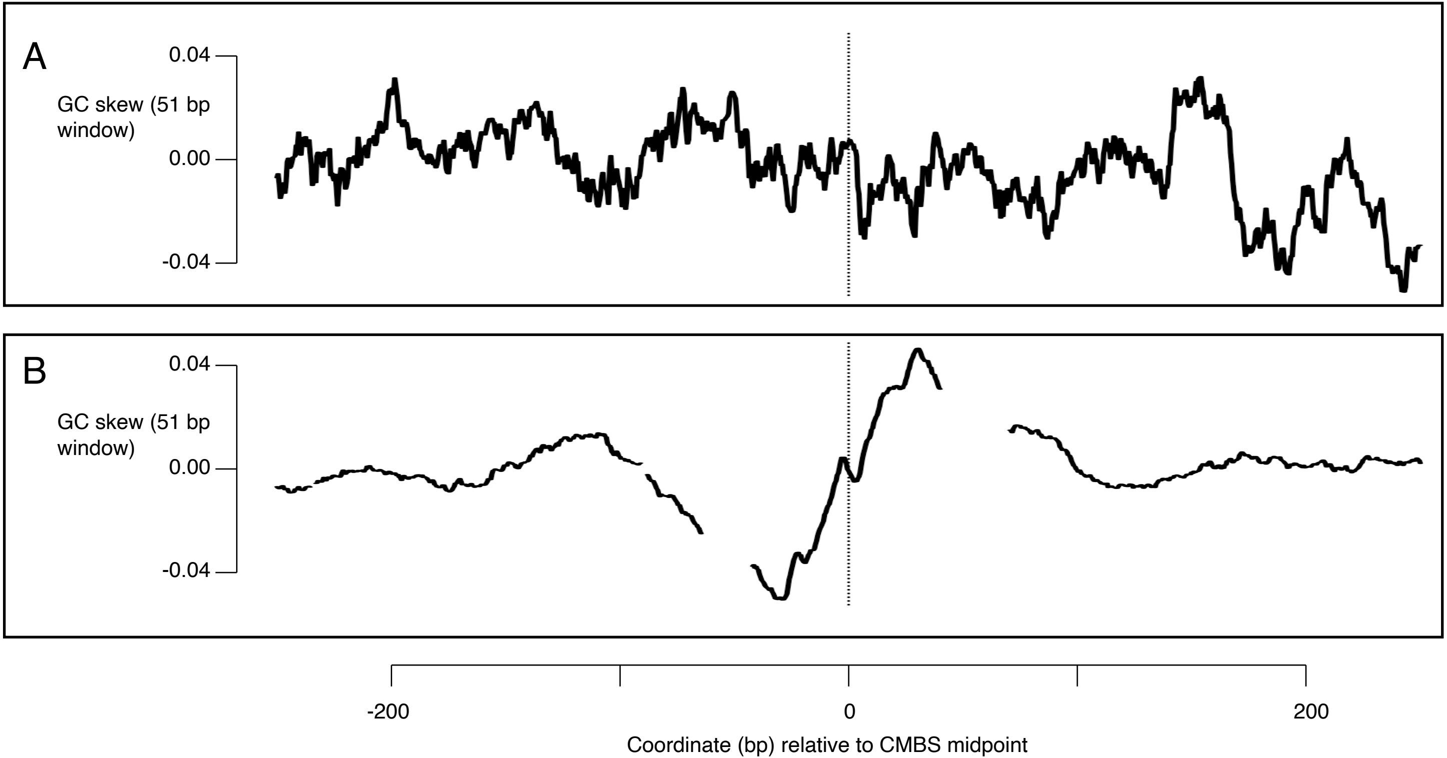
A
0.04
GC skew (51 bp window)
0.00
-0.04
B
0.04
GC skew (51 bp window)
0.00
-0.04
-200
0
200
Coordinate (bp) relative to CMBS midpoint
